# Improving the reports of systematic reviews in sexual medicine

**DOI:** 10.1093/jsxmed/qdae204

**Published:** 2025-02-15

**Authors:** Larissa Shamseer, Ana Patricia Ayala, Andrea C Tricco, Melissa L Rethlefsen

**Affiliations:** Knowledge Translation Program, Li Ka Shing Knowledge Institute, St. Michael’s Hospital, Unity Health Toronto, Toronto, ON, M5B 1W8, Canada; Gerstein Science Information Centre, University of Toronto, Toronto, ON, M5S 1A5, Canada; Knowledge Translation Program, Li Ka Shing Knowledge Institute, St. Michael’s Hospital, Unity Health Toronto, Toronto, ON, M5B 1W8, Canada; Queen’s Collaboration for Health Care Quality: A JBI Centre of Excellence, School of Nursing, Queen’s University, Kingston, ON, K7L 3N6, Canada; Epidemiology Division and Institute for Health, Management, and Evaluation, Dalla Lana School of Public Health, University of Toronto, Toronto, ON M5T 3M7, Canada; Health Sciences Library and Informatics Center, University of New Mexico, MSC 09 5100, 1 University of New Mexico, Albuquerque, NM 87131-0001, USA

**Keywords:** systematic reviews as topic, publishing, review literature as topic, evidence-based medicine, publications, epidemiologic research design

Systematic reviews are the most common type of evidence synthesis in the health care literature, according to a preliminary search of PubMed. They are distinct from literature reviews by nature of using a scientific approach—with methods articulated in advance and with sufficient detail that they can be reproduced by others.^1^ They are a foundational methodology of the overarching “evidence synthesis” category of research design, which collectively encompasses various synthesis methodologies with systematic approaches. This article outlines current problems with the reporting of systematic reviews and suggests tools that authors can use to optimize how systematic reviews are done and reported.

## What is wrong with the reporting of systematic reviews?

According to Cochrane, a world-leading organization in systematic review methodology, a systematic review is defined as, “a review that uses explicit, systematic methods to collate and synthesize findings of a study that address a clearly formulated question”.^2^ Synthesis here, refers to the quantitative synthesis of data from two or more studies, including the descriptive analysis of study characteristics, and may or may not include meta-analysis.^2^ Most research labeled as a “systematic review” does not meet modern definitions of a systematic review.^3^ This reflects both the evolution of evidence synthesis methods^1^ and that many reports labeled as a “systematic review” may omit critical methodological components.^4^^,^^5^ An evaluation of 300 health effectiveness systematic reviews with meta-analyses published in 2020 found suboptimal reporting of several key elements. These include reporting of whether a review is registered (38%), refers to a protocol (5%), key details about search sources (37%) and search strategies (27%), and summary statistics for each included study (72%).^5^

The reporting of systematic reviews in areas of the field of sexual medicine has been characterized as suboptimal, with aspects of literature searches being particularly poor. Only 50% of reviews of Peyronie’s disease describe all information sources, search strategies, and methods of selection of and extraction of data from included studies.^6^ Less than 30% indicated whether the review was registered or had a protocol. The documentation and methods of literature searches in systematic reviews across the urological literature has improved over time, yet is still suboptimal.^7^ Less than 30% of these reviews indicate searching trial registries or other online resources. Only 9% indicated whether included authors were contacted for additional data or studies. This is especially important since data sharing requirements by journals are relatively new and data sharing/availability statements of trials are often missing.^8^

## Why is complete reporting of systematic reviews important?

Systematic reviews have long been regarded as one of the most reliable sources of evidence to inform health decision-making (eg, practice and policy).^9^ They are a valued source of health information for clinicians, patients, researchers, and others (eg, research funders, public, media, industry), often forming the basis of clinical practice guidelines. Systematic review findings are optimally implemented into practice when they are completely, accurately, and transparently reported. When critical information is omitted from review reports, this can have detrimental effects on patient care (Table 1).

**Table 1:** Consequences of incomplete reporting of systematic reviews.

Misinforms treatment decisions	Incomplete or biased reporting of study results (ie, reporting positive or statistically significant outcomes and omitting negative or non-statistically significant results) misrepresents the effectiveness or safety of interventions.^10^ When clinicians unknowingly rely on such evidence to make decisions, patients may receive treatments that are ineffective or potentially harmful.^11^
Delays or leads to problematic implementation of effective interventions	If systematic reviews fail to provide complete details (ie, dose, timing, frequency, route, intervention components) about effective interventions, they may be difficult, if not impossible to replicate in practice.^12^ This is particularly important for complex interventions (ie, those with interacting components^13^).
Patient safety risks	Patients may be exposed to unnecessary risks or adverse effects if systematic reviews fail to report on important safety concerns associated with certain interventions.^14^
Wastes resources	Precious healthcare resources may be misallocated toward interventions that are not supported by robust evidence due to poor reporting. This wastes resources and may divert funding away from safer, more effective treatments or research.^15^
Undermines trust in healthcare	Poor reporting of systematic reviews undermines their credibility and utility in informing evidence-based health decision-making. When exposed, this can lead to skepticism about the reliability of research findings and erode public trust in healthcare systems.^16^

## How can we improve the reporting of reviews?

Reporting guidelines typically include a checklist, flow diagram, and accompanying text developed using explicit methodology aimed at guiding authors on the core reporting elements for a specific type of research.^17^ The Enhancing the QUality And Transparency Of health Research (EQUATOR) Network provides a comprehensive and searchable library of reporting guidelines (https://www.equator-network.org/) that exist for various study designs, including those relevant to systematic reviews and other types of evidence synthesis.

## The PRISMA statement: preferred reporting items for systematic reviews and meta-analyses

For at least 25 years, reporting guidance has existed in some form for systematic reviews. What was originally published as the QUality Of Reports Of Meta-analyses (QUOROM) Statement in 1999^18^ has evolved into the current PRISMA 2020 Statement.^3^ The PRISMA reporting guideline is primarily intended to facilitate reporting of reviews evaluating health care interventions, including quantitative studies (including those where qualitative studies are also present) and addresses the reporting of meta-analyses. While meta-analysis is a feature of almost two-thirds of systematic reviews,^4^ it is not always possible or appropriate (eg, heterogeneous studies/study characteristics, missing outcome data). Such reviews often report their approach to summarizing intervention effects as “descriptive” or “narrative” syntheses; however, descriptions are often haphazard and inconsistent.^19^ For systematic reviews where meta-analyses may not be possible (due to lack of amenable data) but where authors still want to provide a quantitative summary of the data, the Synthesis Without Meta-analysis (SWiM) reporting guideline provides a framework for reporting.^20^ SWiM contains nine reporting items, accompanied by explanations and examples for each. It provides specific guidance for reporting how studies are grouped, synthesis method used (eg, calculating summary statistics of intervention effect estimates, vote counting based on direction of effect, and combining p-values), presentation of data and summary text, and limitations of the synthesis. SWiM explicitly does not apply to narrative syntheses of qualitative data. Specific reporting guidelines for a variety of qualitative data syntheses can be found on the EQUATOR Network.

## Reporting other types of evidence synthesis

At least 40 different types of evidence synthesis exist.^21^  Table 2 outlines a few types of evidence synthesis that have become increasingly common along with their purpose and corresponding reporting guidelines. Researchers carrying out evidence syntheses ought to be mindful in selecting the type of evidence synthesis that best suits their purpose. When investigating interventions (ie, clinical therapeutics) or diagnostics, systematic reviews are best-suited to questions of their effectiveness and accuracy, respectively. In contrast, a scoping review is a suitable way to get an idea of the breadth of evidence available in a particular area. Rapid reviews may be done instances where limited budgets or urgent needs (ie, during a pandemic) exist. However, rapid review authors ought to alert readers to the risks of incomplete evidence and compromised methodological rigor.^30^ Researchers aiming to gain a sense of the scope of literature or to understand the gaps in knowledge on a particular research topic would be best carrying out a scoping review.

**Table 2:** Evidence synthesis types and reporting guidelines.

Type of evidence synthesis	Description and purpose	Relevant reporting guideline
All types of systematic reviews	A review that uses pre-specified methods to collate and synthesize findings of a study that address a clearly formulated question, while minimizing bias. Focuses on providing a precise and comprehensive summary of the evidence related to a well-defined question. Often used to inform clinical practice guidelines, policy decisions, and further research.	PRISMA-P (protocols)^22^^,^^23^ *(currently being updated*^24^*)* PRISMA-S (search)^25^ SWiM^20^ PRISMA 2020^3^
Scoping reviews	A review method used to characterize the breadth of evidence available for a particular topic, field, concept, or issue, often irrespective of source (ie, primary research, reviews, non-empirical evidence) within or across particular contexts.^26^ Often used to identify gaps in knowledge, organize it into groups, clarify concepts, and to inform future research agendas.	PRISMA extension for Scoping Reviews^27^ (*currently being updated*) PRISMA-S (search)^25^
Rapid reviews	A streamlined form of a traditional systematic review, with pre-specified limits on certain methods (ie, time-delimited search) in order to produce evidence in a resource-efficient manner.^28^ Useful for producing a rigorous synthesis for a specific research question when time is of the essence, such as during a health emergency (ie, epidemic or pandemic).	PRISMA extension for rapid reviews (preliminary checklist^29^) PRISMA-S (search)^25^

## How can authors use rigor and transparency to optimize reporting of their reviews?

The rigor and transparency in processes and practices upstream in the review process also factor into what is eventually reported in publications. Comprehensive guidance on systematic review methods have been developed by world experts from leading review organizations and are freely available online. For instance, the Cochrane Handbook offers guidance on systematic reviews of health interventions^2^; the JBI Manual for evidence synthesis offers methodological guidance for both systematic and scoping reviews.^31^ Additionally, these organizations regularly offer review training courses, found on their websites. Researchers ought to consult these resources prior to embarking on a review, while drafting a protocol, and throughout the review process.

Based on the experience of our author group in writing, conducting, and developing reporting guidelines for various evidence syntheses methods, we have identified five broad principles that researchers ought to adhere to during the review process to optimize their written report. We have also developed an accompanying figure outlining basic steps in the systematic review process with corresponding tools for facilitating rigor and transparency.

## Identify the appropriate review methodology

Before embarking on a review, researchers ought to ensure that that method of evidence synthesis is appropriate for answering the review question. To do so, researchers could utilize the freely available *Right Review* tool (https://rightreview.knowledgetranslation.net), designed to assist users in identifying an evidence synthesis method that best-suited to their research question. The tool currently covers 26 methods for quantitative evidence syntheses and 15 qualitative evidence synthesis methods and is currently being updated.^32^^,^^33^ It queries users on aspects of their intended review and outputs the most relevant evidence synthesis method.

## Engage an information specialist at review outset

Information specialists and librarians are critical members of a systematic review team. Their involvement as review co-authors is associated with higher quality search strategies.^34^ Reaching out to an information specialist at the start of a review project can save significant time and bring pertinent expertise to the review. For instance, at the outset of a review, information specialists can help develop the review question by clarifying the end goal of the review, ensuring that the review will not be duplicative, and identifying whether evidence to answer the review question will be available.^35^ They will identify the appropriate databases to search to answer the review question and, most important, they will conduct the sensitive, comprehensive, and reproducible searches needed for a systematic review. A key step in this type of search is to peer review the search prior to conducting it, usually by another information specialist using a tool called PRESS, or Peer Review of Electronic Search Strategies.^36^ This peer review improves the search quality and reduces the potential for errors or missing terminology. Documenting and reporting these searches transparently for publication is another role for information specialists who will be highly familiar with PRISMA 2020 and its search-related extension, PRISMA-Search.^25^

Though not all researchers will have access to a skilled information specialist, researchers at academic institutions and hospitals are likely to have such support freely available within their institution. In addition, fee-based consultation services are also available from some specialists. Authors who work with established review organizations such as Cochrane, Campbell, or JBI have free access to information specialists within these organizations.

## Develop and register a review protocol

Once a review question and corresponding review methodology are identified, researchers ought to create a protocol to guide decision-making during the review process (eg, selection criteria, data extraction and transformation, and risk of bias assessments). The PRISMA extension for protocols (PRISMA-P) provides guidance for documenting planned methods and analyses of systematic reviews in the form of a 17-item checklist^22^ and an elaboration document containing explanations and examples for each checklist item.^23^ Working from a protocol helps prevent researcher biases from entering the review process and facilitates consistency in decision-making.

When protocols are made readily available, they can optimize transparency in the review process, help readers evaluate the potential for bias (ie, selective reporting), and reduce unintended duplication of efforts. The International Prospective Register of Systematic Reviews (PROSPERO) enables researchers to register key methodological details before carrying out the systematic review, and to attach a full protocol to the registration record registry (https://www.crd.york.ac.uk/prospero/).^37^ Several other registries exist in which to register protocols for other types of evidence synthesis, such as scoping reviews, including the Open Science Framework: (https://help.osf.io/article/330-welcome-to-registrations). Some journals also publish protocols as open access articles for a fee (eg, BMJ Open, Systematic Reviews); some review organizations published review protocols as a mandatory first step (eg, Cochrane or the Campbell Collaboration).

Authors ought to consider including a statement in their protocols signaling their use of PRISMA-P and indicate where it is registered. For example, “This protocol has been prepared in accordance with the PRISMA-P checklist [insert citation] and is registered at [insert registration URL]”.

## Use tools and technology to facilitate and track review processes

**Figure 1:**
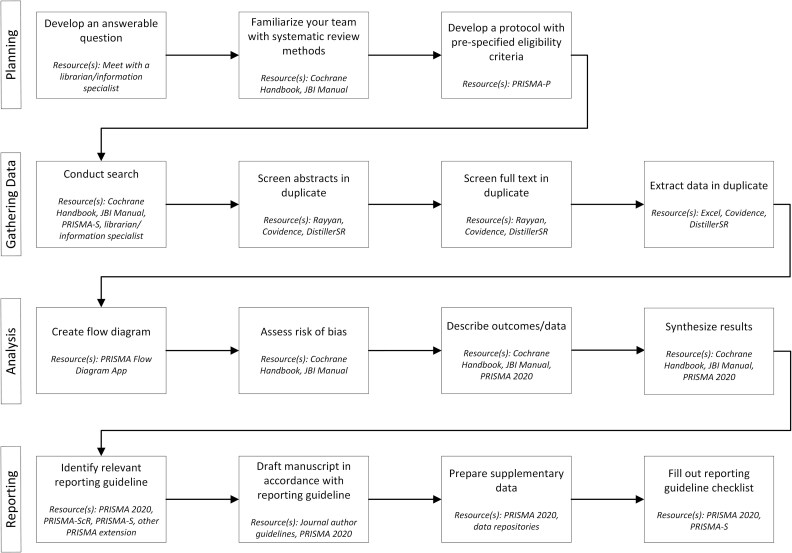
Essential systematic review process and resources.

Many tools and technologies have become available over the last decade, specifically to facilitate review conduct and reporting. While they are too numerous to list here, we have provided some examples of review software in Fig. 1. Utilizing such technologies ought to expedite the review process while facilitating transparent tracking of records and decisions throughout the review process. For instance, while it is possible and acceptable to carry out reviews in Excel, specialized systematic review software exists to help manage, screen, extract data, and track review records (eg, Rayyan, Covidence, DistillerSR). Researchers should be mindful of the risks, accuracy, and journal policies of using artificial intelligence (AI) to complete screening and data extraction in reviews, and report the use of AI in review manuscripts as per item 9 of the PRISMA 2020 checklist.^3^ Tracking the inclusion and exclusion of review records and reasons why, from the initial search through to data analysis is critical to completing a PRISMA flow diagram. Some of this software is freely available online, may be freely accessible through subscriptions with institutional libraries, or may be cheaply accessible for certain under-resourced groups. Some technologies, such as those for completing PRISMA flow diagrams (https://estech.shinyapps.io/prisma_flowdiagram/), predicting the time requirements for a systematic review (https://predicter.github.io/), and many others, are freely available from Evidence Synthesis Hackathon (https://www.eshackathon.org/).

## Use PRISMA 2020 and its extensions

Researchers should use the relevant reporting checklist from the outset of the review writing process. In addition to the 27-items of the PRISMA 2020 checklist,^3^ authors should consult the PRISMA-S checklist to completely document search methods in all systematic reviews. The accompanying “Explanation and Elaboration” articles^38^ are key resources for authors with examples of how each item ought to be reported. Authors may fill-out a PRISMA checklist manually or using an online user-friendly interface: https://prisma.shinyapps.io/checklist/. Completed checklists may be requested by journals to assist in peer review.^39^ PRISMA 2020 also provides a 10-item guide for reporting review abstracts. Additionally, as many extensions and complements to PRISMA exist (ie, for reviews using individual patient data,^40^ or conducting network meta-analyses^41^), authors ought to consult the PRISMA website to identify any that are applicable to their review. A tool is currently under development to integrating PRISMA and its extensions to create customized checklists according to review design.^42^ Additionally, as per PRISMA item 27, authors ought to include a statement about the public availability of review data and code. Authors might consider engaging a statistician or author with statistical expertise at the outset of the review project to ensure appropriate statistical techniques are used and reported, and that data and code are in a shareable format once the review is completed.

Authors ought to consider including a statement in their review manuscripts signaling their use of PRISMA or a relevant extension or guideline and again indicate where the review is registered. For example, “This review is reported in accordance with the PRISMA 2020 checklist [insert citation] and is registered at [insert registration URL]”.

The use of reporting guidelines is associated with more complete reporting across a variety of study designs. A 2014 meta-analysis showed that journals that endorse authors’ use of PRISMA within their *Instructions to Authors* publish systematic reviews that completely report more items of the PRISMA checklist than those that do not.^43^ Journals may uphold their own processes and requirements regarding PRISMA, and study reporting more generally. Authors ought to consult a journals’ Instructions to Authors before submitting and adhere to any relevant policies. At a minimum, authors ought to follow the 5 principles outlined here and the review process in Fig. 1 to facilitate the review process, optimize their chances of being published, and facilitate maximum usability by readers.
